# Hybrid asexuality as a primary postzygotic barrier between nascent species: On the interconnection between asexuality, hybridization and speciation

**DOI:** 10.1111/mec.14377

**Published:** 2017-11-29

**Authors:** Karel Janko, Jan Pačes, Hilde Wilkinson‐Herbots, Rui J. Costa, Jan Roslein, Pavel Drozd, Nataliia Iakovenko, Jakub Rídl, Miluše Hroudová, Jan Kočí, Radka Reifová, Věra Šlechtová, Lukáš Choleva

**Affiliations:** ^1^ Institute of Animal Physiology and Genetics Laboratory of Fish Genetics The Czech Academy of Sciences Libechov Czech Republic; ^2^ Department of Biology and Ecology Faculty of Science University of Ostrava Ostrava Czech Republic; ^3^ Institute of Molecular Genetics Laboratory of Genomics and Bioinformatics The Czech Academy of Sciences Prague Czech Republic; ^4^ Department of Statistical Science University College London London UK; ^5^ Department of Fish Ecology Institute of Vertebrate Biology The Czech Academy of Sciences Brno Czech Republic; ^6^ Schmalhausen Institute of Zoology of NAS of Ukraine Kyiv Ukraine; ^7^ Department of Zoology Faculty of Science Charles University Prague Czech Republic

**Keywords:** balance hypothesis, coalescence, evolution of asexuality, hybridization, phylogeography, speciation

## Abstract

Although sexual reproduction is ubiquitous throughout nature, the molecular machinery behind it has been repeatedly disrupted during evolution, leading to the emergence of asexual lineages in all eukaryotic phyla. Despite intensive research, little is known about what causes the switch from sexual reproduction to asexuality. Interspecific hybridization is one of the candidate explanations, but the reasons for the apparent association between hybridization and asexuality remain unclear. In this study, we combined cross‐breeding experiments with population genetic and phylogenomic approaches to reveal the history of speciation and asexuality evolution in European spined loaches (*Cobitis*). Contemporary species readily hybridize in hybrid zones, but produce infertile males and fertile but clonally reproducing females that cannot mediate introgressions. However, our analysis of exome data indicates that intensive gene flow between species has occurred in the past. Crossings among species with various genetic distances showed that, while distantly related species produced asexual females and sterile males, closely related species produce sexually reproducing hybrids of both sexes. Our results suggest that hybridization leads to sexual hybrids at the initial stages of speciation, but as the species diverge further, the gradual accumulation of reproductive incompatibilities between species could distort their gametogenesis towards asexuality. Interestingly, comparative analysis of published data revealed that hybrid asexuality generally evolves at lower genetic divergences than hybrid sterility or inviability. Given that hybrid asexuality effectively restricts gene flow, it may establish a primary reproductive barrier earlier during diversification than other “classical” forms of postzygotic incompatibilities. Hybrid asexuality may thus indirectly contribute to the speciation process.

## 1 INTRODUCTION

Sexual reproduction is one of the most ubiquitous properties of eukaryotes. However, although the underlying molecular machinery is highly conserved, it has been repeatedly disrupted in many different ways, leading to independent emergences of asexual lineages occurring in all phyla (Neiman, Sharbel, & Schwander, 2014). Eukaryotic lineages collectively termed as “asexual” are scattered all over the tree of life and employ a wide spectrum of independently arisen cytological mechanisms for gametes production, which can considerably differ even between closely related asexual taxa (Stenberg & Saura, 2009, 2013). Although the reasons for the loss of sex have been studied for over a century, rev. in Carman (1997), the answer remains elusive apart from some straightforward cases, such as *Wolbachia*‐induced asexuality (e.g., Pike & Kingcombe, 2009), leaving us with various candidate hypotheses.

The “asexuality mutation” hypothesis assumes relatively simple loss‐of‐function mutations (e.g., Eads, Tsuchiya, Andrews, Lynch, & Zolan, 2012; Mogie, 1992) and is especially appealing for facultative and/or cyclical asexuals, which are already predisposed to produce asexual gametes. The *phylogenetic‐constraint hypothesis* highlights the observation that asexuality prevails only in some taxa (Hotz et al., 1985; Murphy, Fu, Macculloch, Darevsky, & Kupriyanova, 2000), indicating that some phylogenetic lineages have “predispositions” for uniparental reproduction (e.g., inherent production of unreduced gametes in low levels (e.g., Aliyu, Schranz, & Sharbel, 2010), while others are deprived of such possibility (e.g., due to sex‐specific imprinting of genes (Kono et al., 2004)).

An alternative class of theories accentuates the *hybrid origin* of many, if not the absolute majority of asexual animals (Neaves & Baumann, 2011; Simon, Delmotte, Rispe, & Crease, 2003). In spite of intensive research, it is still unclear whether there are any general rules how interspecific hybridization initiates asexual reproduction. However, already a century ago, Ernst (1918) hypothesized that outcomes of hybridization may follow a continuum from fully sexual to obligately asexually reproducing hybrids depending on how closely related the hybridizing species are. Current opinions differ on if and why the likelihood of hybrid asexuality should depend on genetic distance between hybridizing genomes. Having noted that the proportion of unreduced gametes is larger in hybrids between distantly related rather than closely related species, Moritz et al. (1989) formulated the “balance hypothesis.” It postulates that asexuality results from accumulation of incompatibilities between hybridizing species that disrupt cellular regulation of sexual reproduction. Hybrid asexuality can thus arise only when the genomes of parental species are divergent enough to disrupt meiosis in hybrids, yet not divergent enough to seriously compromise hybrid viability or fertility. De Storme and Mason (2014) suggested that unreduced gametes might result from hampered pairings of homologues due to decreasing sequence homology among divergent hybridizing species. Carman (1997) suggested that rather than a mere consequence of accumulated genetic divergence, the asexuality results from asynchronous expression of genes brought together by hybridization between species with differently timed developmental programs.

Unfortunately, empirical support for the aforementioned models is rather scarce (Dijk, 2009). For example, the parental species of some hybrid asexuals appear to be phylogenetically distant relatives to each other rather than being sister species (e.g., Jančúchová‐Lásková, Landová, & Frynta, 2015; Moritz, Densmore, Wright, & Brown, 1992; Moritz, Uzzell, et al., 1992; Moritz, Wright, & Brown, 1992), but others argued that such phylogenetic patterns may still be explained by the phylogenetic‐constraint hypothesis (Hotz et al., 1985; Murphy et al., 2000). After all, the causal role of hybridization in asexuality induction remains speculative (Kearney, Fujita, & Ridenour, 2009), as most attempts to experimentally synthesize a new asexual lineage from a crossing of sexual species failed (reviewed by Choleva et al., 2012). Ultimately, it is not understood why hybridization should affect meiosis in a similar way across diverse taxa (Kearney et al., 2009). In this study, we suggest that general causality interconnecting hybridization and asexuality does exist and may be even more integral than previously believed.

A pervasive observation in speciation literature is that hybridization capability decreases as one moves from closely to distantly related pairs of taxa (Kropáčková, Piálek, Gergelits, Forejt, & Reifová, 2015; Russell, 2003; Rykena, 2002; Sánchez‐Guillén, Córdoba‐Aguilar, Cordero‐Rivera, & Wellenreuther, 2014), which is believed to result from gradual accumulation of genetic incompatibilities that cause intrinsic postzygotic isolation, that is, hybrid infertility and inviability (rev. in Seehausen et al., 2014). While the rate at which intrinsic postzygotic reproductive isolation mechanisms (RIMs) accumulate appears nonlinear and taxon‐specific (Bolnick & Near, 2005; Edmands, 2002; Matute, Butler, Turissini, & Coyne, 2010; Orr & Turelli, 2001), hybrid infertility generally evolves at lower genetic distances than hybrid inviability (Price & Bouvier, 2002; Russell, 2003) and generally affects the heterogametic sex earlier in evolution (Haldane, 1922). It is interesting to note that Moritz et al. (1989) also hypothesized that asexual gametogenesis in hybrids may relate to the amount of divergence accumulated across many genes, rather than to the presence of a specific allele at some particular locus. If so, hybrid asexuality may be viewed as a special case of the accumulation of Dobzhansky–Muller incompatibilities that disrupt critical processes—sexual reproduction in this case. Such a view also implies an interesting perspective on which we elaborate in this study: at early stages of the species diversification process when hybrids' fitness has not yet considerably decreased, accumulated incompatibilities may distort hybrids' reproductive mode towards asexuality, and because clonal transmission of hybrids' genomes prevents interspecific gene exchange (e.g., Keller, Wolinska, Tellenbach, & Spaak, 2007; Lampert et al., 2007), such a change may in turn contribute to speciation.

**Figure 1:**
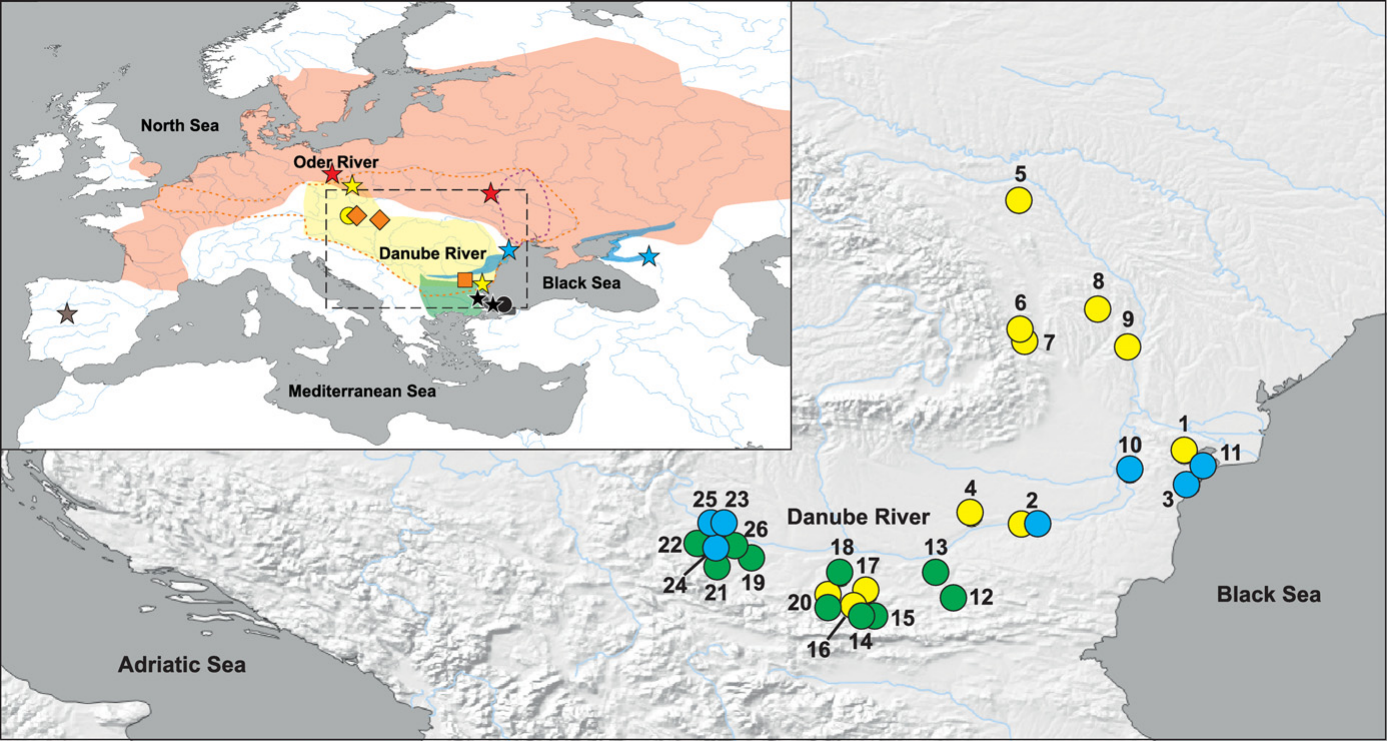
Map of sampling sites and distribution of species and hybrid biotypes. The inset shows the European distribution of the sexual species studied. Red stands for the *Cobitis taenia* distribution range, yellow for *C. elongatoides*, blue for *C. tanaitica*, black for *C. pontica* and green for *C. strumicae*. The orange dotted line delimits the distribution of the ancient clonal lineage, the so‐called hybrid clade I, the purple dotted line the distribution of the so‐called hybrid clade II. Stars indicate sampling sites of individuals used for exome‐capture analyses (the *C. paludica* outgroup is in brown), circles, squares and diamonds those used in crossing experiments (circles indicate sampled sexual species, the orange square stands for diploid and diamonds for triploid *C*. *elongatoides–tanaitica* hybrids used in crossings). The main map shows in detail the Lower Danubian hybrid zone: yellow circles indicate localities with *C. elongatoides*, blue represents *C. tanaitica*, and green indicates *C. strumicae*. Locality numbers correspond to Table S1

We explore the interconnection between hybrid asexuality and speciation by reconstructing the initiation of asexuality and the diversification history of European spined loaches (*Cobitis*). There are several European *Cobitis* lineages sensu Bohlen, Perdices, Doadrio, and Economidis (2006), of which one comprises several morphologically and ecologically very similar species that have parapatric distribution and meet in hybrid zones. Among these, four closely related species (*C. tanaitica*,* C. taenia*,* C. taurica* and *C. pontica*) have Ponto‐Caspian distribution (*C. taenia* further colonized northern and western Europe) while their distant relative, *C. elongatoides*, is distributed throughout the Danubian Basin. Their ranges have been fluctuating over the Quaternary (Janko, Culling, Rab, & Kotlik, 2005) and overlap in central Europe, the Lower Danube Basin and Southern Ukraine (Janko, Flajšhans, et al., 2007) (Figure 1). Hybridization has been documented to take place in these zones, and reproductive contact between *C. elongatoides* and either *C. taenia*,* C. tanaitica* or *C. pontica* results in clonally reproducing all‐females hybrid lineages (Choleva et al., 2012; Janko, Bohlen, et al., 2007; Janko, Flajšhans, et al., 2007). These hybrid lineages achieved remarkable evolutionary success and colonized most of the European continent, some of them having achieved considerable ages (i.e., the so‐called hybrid clades I and II are as ancient as 0.35 and 0.25 Mya, respectively (Janko et al., 2005, 2012)). Clonal reproduction of these hybrid lineages theoretically prevents any interspecific gene flow between extant species, but Choleva et al. (2014) documented evidence for historical replacement of the original *C. tanaitica* mitochondrion by a *C*. *elongatoides*‐like mitochondrion. This suggests that introgressive hybridization has been possible in the past.

To understand the speciation history and the evolution of hybrid asexuality during fish speciation, we employed multiple complementary approaches examining (i) whether the formation of asexual hybrids does depend on genetic distance between parental species and (ii) whether the establishment of reproductive barriers can be primarily accomplished by the formation of hybrid asexuality. First, we analysed the reproductive modes in hybrids between several differently related *Cobitis* species to reveal their asexuality and the extent of the currently observed reproductive isolation. Second, we performed a detailed population genetic analysis of the *elongatoides – taenia* (Janko et al., 2012) and *elongatoides – taenia* (this study) hybrid zones to test whether there is any ongoing introgressive hybridization. Third, we employed exomewide coalescent analyses to estimate the levels and timing of historical gene flow among species. Finally, we tested the generality of our hypothesis by literature surveys on published cases of hybrid and/or asexual fish. The results implied that *Cobitis* diversification has indeed been accompanied by decreasing intensity of introgressive hybridization. However, unlike “classical” speciation cases, such a restriction in gene flow has not been accompanied by evolution of classical RIMs but rather by the production of asexual hybrids.

## 2 MATERIALS AND METHODS

To test whether the formation of asexual hybrids depends on the genetic distance between parental species and how the formation of hybrid asexuality affects interspecific gene flow, we performed the following four analyses.

### 2.1 Analysis of reproductive modes of hybrids

In the first experiment, we analysed reproductive modes of naturally occurring diploid and triploid hybrid EN and EEN females (Table 1; letters E and N stand for haploid *elongatoides* and *tanaitica* genomes, respectively) by crossing them with sexual males following (Choleva et al., 2012). The families were genotyped using the microsatellite multiplex 1 with loci Cota 006, Cota 010, Cota 027, Cota 032, Cota 068, Cota 093 and Cota 111 (Choleva et al., 2012; De Gelas, Janko, Volckaert, De Charleroy, & Van Houdt, 2008) to verify their gynogenetic reproduction. We further performed allozyme analysis (Janko, Flajšhans, et al., 2007) to compare profiles of individual eggs to maternal somatic tissues to test for hybridogenetic type of reproduction. During hybridogenesis, reduced, albeit nonsegregating gametes are produced by premeiotic exclusion of one parent's genome leading to protein expression of only one parental taxon per egg (Carmona, Sanjur, Doadrio, Machordom, & Vrijenhoek, 1997; Cimino, 1972; Uzzell, Hotz, & Berger, 1980).

**Table 1:** Crossing experiments

Female ID	Biotype	Origin	Male ID	Biotype	Origin	Progeny			Family ID
						Sexual	Clonal	Polyploid	
EENF1	EEN	Okna R., Slovakia	EEM1	EE	Okna R., Slovakia	0	6	1	No. 401
EENF24	EEN	Okna R., Slovakia	EEM8	EE	Okna R., Slovakia	0	16	0	No. 424
EENF25	EEN	Okna R., Slovakia	EEM8	EE	Okna R., Slovakia	0	9	0	No. 425
EENF10	EEN	Ipeľ R., Slovakia	EEM9	EE	Nová Říše, Czech R.	0	9	0	No. 413
097CENNF1	EN	Jantra R., Bulgaria	09EXPM7C	PP	Veleka R., Bulgaria	0	6	4	No. 8
10EXF1F9C08	TP	Laboratory hybrid	10EXF1M9C08	TP	Laboratory hybrid	11	6	9	No. 1
F1TFPM062	TP	Laboratory hybrid	F1TFPM065	TP	Laboratory hybrid	6	0	0	No. 17; clutch A
F1TFPM062	TP	Laboratory hybrid	F1TFPM066	TP	Laboratory hybrid	1	0	0	No. 17; clutch B

Biotype—E, haploid *Cobitis elongatoides* genome; N, haploid *C. tanaitica* genome; P, haploid *C. pontica* genome; T, haploid *C. taenia* genome. For each family, we indicate different types of progeny: “sexual” denotes a number of progeny obtained from segregating gametes; “clonal,” a number of progeny obtained from clonal gametes; “polyploid,” a number of progeny obtained from fertilized clonal gametes. Note that several clutches from different F1 individuals occurred in the experimental family No. 17. GPS coordinates: Okna R. 48.718100, 22.120511; Ipeľ R. 48.072868, 19.088204; Nová Říše 49.152760, 15.547374; Jantra R. 43.469008, 25.725494; Veleka R. 42.026909, 27.623940.

In the second experiment, we individually crossed *C. taenia* with *C. pontica*, reared their F1 progeny until sexual maturity and produced the F2 generation. The families were genotyped as described above.

### 2.2 Analysis of *C. elongatoides*–*C. tanaitica* hybrid zone and test of ongoing introgressive hybridization

Specimens captured at the Lower Danubian hybrid zone (Figure 1, Table S1) were first classified into taxonomic units using allozyme and PCR‐RFLP diagnostic markers (Janko, Flajšhans, et al., 2007) and their ploidy examined by flow cytometry. All diploids were genotyped with microsatellite multiplex 1 and the locus Cota 033. We checked the microsatellite data in microchecker version 2.2.3 (Van Oosterhout, Hutchinson, Wills, & Shipley, 2004) for null alleles, large allele dropouts and scoring errors. Summary statistics and deviations from Hardy–Weinberg equilibrium (HWE), *F*_ST_ and pairwise linkage equilibria were evaluated for each locus per taxon using msa version 4.05 (Dieringer & Schlötterer, 2003), genepop version 4.1.3 (Rousset, 2008) and fstat version 2.9.2 (Goudet, Perrin, & Waser, 2002). The GenAlEx 6.5 software (Peakall & Smouse, 2006) was used to identify all unique multilocus genotypes (MLG), and we employed the GenClone software (Arnaud‐Haond & Belkhir, 2007) to calculate the probability that observed multiple copies of the same MLG arose by independent sexual events (*P*_SEX_), taking into account the deviations from HWE (*P*_GEN(FIS)_; Arnaud‐Haond, Duarte, Alberto, & Serrao, 2007). As described in Janko et al. (2012), we further identified groups of MLGs that are related to each other more closely than expected by chance alone and might therefore represent members of the same clone, the so‐called multilocus lineage (MLL). We calculated the sum of the differences in allele lengths between each pair of MLG (Meirmans & Van Tienderen, 2004). Subsequently, we simulated hybrid genotypes by 10,000 random combinations of individuals from hybridizing species to obtain the null distance distribution, which was used to evaluate the probability that any two MLG belong to the same clone after correcting for multiple comparisons with the sequential Bonferroni's correction.

We subsequently sequenced a 1,188‐bp fragment of the cytochrome *b* gene according to Janko, Flajšhans, et al. (2007) in a subset of diploid individuals and used the median‐joining network (Bandelt, Forster, & Röhl, 1999) drawn with NETWORK (http://www.fluxus-engineering.com/netwinfo.htm) to put newly discovered haplotypes into the context of previously published mtDNA variability (Choleva, Apostolou, Ráb, & Janko, 2008; Janko et al., 2012).

Combined microsatellite and allozyme data were used to detect admixed diploid individuals by two clustering methods. We first used the admixture model implemented in Structure 2.3.4 (Falush, Stephens, & Pritchard, 2003; Pritchard, Stephens, & Donnelly, 2000) to compute the parameter *q*, that is, the proportion of an individual's genome originating from one of the two inferred clusters, corresponding to the parental species *C. elongatoides* and C*. tanaitica*. The analysis was based on runs with 10^6^ iterations, following a burn‐in period of 5 × 10^4^ iterations. Three independent runs for the number of populations varying from *K = *1 to *K = *10 were performed, and the best value of *K* was chosen following Evanno, Regnaut, and Goudet (2005), with Structure Harvester (Earl & vonHoldt, 2011).

The Bayesian clustering method implemented in NewHybrids 1.1 (Anderson & Thompson, 2002) was also used to compute the posterior probability that an individual belongs to one of the eight predefined classes: *C. elongatoides*,* C. tanaitica*, F1 hybrid, F2 hybrid, and two types of backcross to either *C. elongatoides* or *C. tanaitica*. The two backcross types included those having 75% of their genome originated from the backcrossing species (B1 generation) and those having 95% of their genome originated from the backcrossing species (further‐generation backcrosses). Posterior distributions were evaluated by running five independent analyses to confirm convergence. We started with different random seeds, performed 10^4^ burn‐in iterations and continued with 500,000 Markov chain Monte Carlo iterations without using prior allele frequency information. Analyses were run for four combinations of prior distributions (uniform or Jeffreys for Ɵ and π parameters) to explore the robustness of the results (Anderson & Thompson, 2002).

To minimize the effect of clonal propagation, we used only one randomly chosen representative of each unique MLL and repeated the analysis several times to check its robustness against the particular choice of MLL representatives (Janko et al., 2012). We also repeated those analyses with either the Cota_006 or Cota_041 loci removed due to their possible linkage (De Gelas et al., 2008).

### 2.3 Estimation of levels and timing of historical gene flow among the species using exomewide SNP data

#### 2.3.1 Assembly of exome reference from RNAseq data of nonmodel fish species

The mRNA sequencing concerned liver tissues of six *C. taenia* individuals and also oocyte tissue from one of them. Isolated RNA was transcribed into cDNA, normalized by Trimmer cDNA normalization Kit (Evrogen, Moscow, Russia) and sequenced using GS FLX+ chemistry (454 Life Sciences, Roche). The initial 1,886,536 reads from *C. taenia* were quality filtered and trimmed from adaptors and primer sequences using Trimmomatic software (Bolger, Lohse, & Usadel, 2014). Technical PCR multiplicates were removed using cdhit‐454 software (Niu, Fu, Sun, & Li, 2010). The resulting 1,707,769 reads (568,470,258 base pairs) were used for assembly using Newbler (Software Release: 2.6 20110517_1502) with parameters: read length >40; overlap length >40; match: >90%; contig length >300 bp. To minimize the potential effect of undetected paralogy, copy number variation, repetitions and misassemblies, we removed all contigs where subsequent read mapping was not unique. The final assembled transcriptome consisted of 20,385 potential mRNAs (average length 1,096.5 bp, total length 22,355,325 bp and N50 1,246 bp; Data available from the Dryad Digital Repository).

#### 2.3.2 DNA isolation and exome‐capture procedure

To extract the information about SNP variability of homologous loci from previously collected ethanol‐preserved fish material, we used the assembled transcriptome to design the probes for targeted enrichment of gDNA loci. 7,000 contigs with highest coverage and assembly quality were sent to NIMBLEGEN for probe design, so that the total length of all contigs is less than 10 Mbp according to the manufacturer's suggestions. We used the probes to obtain enriched exome libraries from stored tissue material of two individuals of each *C. taenia*,* C. elongatoides*,* C. tanaitica* and *C. pontica* (Figure 1; data available from the Dryad Digital Repository) as well as of *C. paludica*—the outgroup species from the Iberian Peninsula, collected in the *terra typicae*. High molecular gDNA was fragmented with a Bioruptor (NextGen, Diagenode, Liege, Belgium) to obtain the required fragment length. DNA libraries with appropriate barcodes were prepared with the KAPA Library Preparation kit for Illumina platforms (KAPA Biosystems), and we followed the NimbleGen SeqCap EZ Library SR protocol (Roche) for sample mixing, hybridization with probes, captured DNA recovery and amplification. Libraries were sequenced with Illumina HiSeq 100‐bp paired‐end reads.

An additional four samples of two C. *elongatoides–taenia* F1 hybrid individuals and both their parents (data available from the Dryad Digital Repository) following the design of a previous crossing experiment (Choleva et al., 2012) were sequenced to experimentally verify the efficiency of exome capture and SNP calling by parent‐to‐offspring comparison.

#### 2.3.3 SNP calling

The obtained fastq files were trimmed based on quality by the fqtrim tool (Pertea, 2015) with the following parameters: minimum read length 20 bp; 3′ end trimming if quality drops below 15; polyA/T trimming was not performed as no homopolymers were enriched in reads. After aligning reads to the reference transcriptome with the BWA MEM algorithm (Li & Durbin, 2009), and processing the resulting files with Picard tools version 1.140 (Broad Institute, http://broadinstitute.github.io/picard), we applied gatk version 3.4 best practices recommendations (DePristo et al., 2011; McKenna et al., 2010; Van der Auwera et al., 2013) for individuals' variants calling. Each individual's variants were called with the HaplotypeCaller tool, and then, all individuals were jointly genotyped using the GenotypeGVCFs tool. For variant recalibration, we used our own SNP database from our ongoing RNAseq study of *C. elongatoides*,* C. taenia* and hybrid females (unpublished Master's Thesis https://is.cuni.cz/webapps/zzp/detail/168590/). We selected species‐diagnostic positions, where all specimens from both hybridizing species were fixed for alternative alleles and all hybrids were heterozygous, to create a learning set for variant quality score recalibration tool VariantRecalibrator and employed it to exome‐capture data. Variants were then filtered with the ApplyRecalibration tool using several tranche sensitivity thresholds. After inspection of the data, we applied the 99.5% tranche to filter all variants. All resulting highly confident SNPs with coverage >15 were transferred into the database using our own SQL scripts, and we ultimately sorted the identified SNPs from each locus into individual locus‐specific matrices (Appendix S1) for subsequent model‐based analyses.

We further identified potential paralogs using the same rationale as in Gayral et al. (2013). To do so, we identified excessively heterozygous contigs, which possessed positions where identical heterozygotic states occurred in all ingroup species as well as in the distant outgroup (*C. paludica*). Such spurious heterozygote calling is unlikely to result from common biological processes but rather from mapping of reads from undetected paralogs, and so we excluded such loci from subsequent analyses.

#### 2.3.4 Reconstruction of historical gene flow

Detection of interspecific gene flow from SNP data was based on recently introduced coalescent‐based maximum‐likelihood methods (Costa & Wilkinson‐Herbots, 2016, 2017; Wilkinson‐Herbots, 2008, 2012, 2015), which are computationally inexpensive and estimate simultaneously the population sizes and migration rates as well as population splitting times, including some scenarios of time‐variable migration rates.

**Figure 2:**
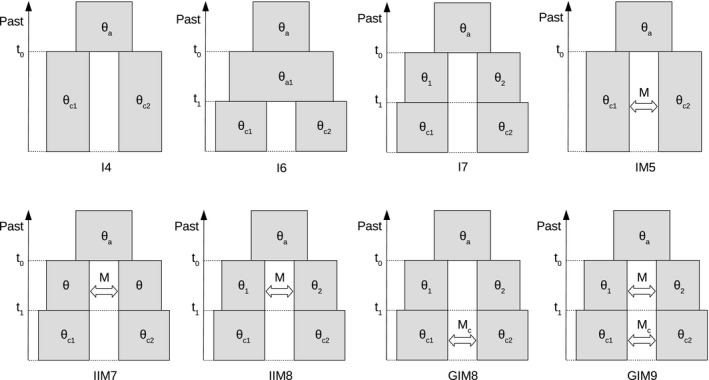
Schematic view of the eight coalescent models. Arrows along the side of model diagrams indicate the respective time periods. The population size parameter is defined as Ɵ_*i*_ *= *4*N*_*i*_μ, where *N*_*i*_ is the effective diploid size of species *i* and μ is the mutation rate per sequence per generation, averaged over the loci included in the analysis; the migration rate is defined as *M = *4*Nm*, where *m* is the proportion of migrants per generation. Wherever an index “c” accompanies the parameter name, it will always indicate the values relevant for current populations, while an index “a” indicates the states of ancestral populations before the split

Contigs matching to mitochondrion were identified by blasting against *Cobitis takatsuensis* mtDNA (GenBank Accession no. NC_015306.1) and removed from analysis. The remaining nuclear data were used to fit the following types of models, which are characterized in Figure 2:

1. The strict isolation models (labelled I4, I6 and I7) with four, six or seven parameters, respectively, assuming that an ancestral population split into two isolated descendant populations. The I6 and I7 models allow for one additional change in size of either the ancestral or the descendant populations, respectively;
2. The “isolation‐with‐migration” model (IM5) with five parameters, where an ancestral population of size Ɵ_a_ split at time *t*_0_ into two descendant populations of equal (IM4) or unequal (IM5) sizes interconnected by gene flow at rate *M*_c_;
3. The “isolation‐with‐initial‐migration” models (IIM7 and IIM8) with seven or eight parameters, respectively, assuming that an ancestral population of size Ɵ_a_ split at time *t*_0_ into two descendant populations (of equal or unequal sizes depending on the model) interconnected by gene flow at rate *M*, lasting until time *t*_1_ after which two descendant populations of sizes Ɵ_c1_ and Ɵ_c2_ evolved in isolation until the present;
4. The most complex “generalized isolation‐with‐migration” models were employed in two variants. The one with nine parameters (GIM9) assumes that an ancestral population of size Ɵ_a_ split at time *t*_0_ into two descendant populations of sizes Ɵ_1_ and Ɵ_2_ interconnected by gene flow at rate *M* until *t*_1_, from which time onwards both species are at their current sizes Ɵ_c1_ and Ɵ_c2_ and gene flow occurs at its current rate *M*_c_. The other model with eight parameters (GIM8) assumed *M* = 0 and modelled the secondary contact scenario. Note that all models described above are nested within the GIM9 model.

All models were ranked according to their Akaike Information Criterion (AIC) score, and we also calculated the evidence ratios providing a relative measure of how much less likely a given model is compared to the best‐fitting model, given the set of candidate models considered and the data (Anderson, 2008); Table S3. Likelihood ratio tests were performed to compare pairs of nested models, where, in the case of parameters on the boundary, we used the appropriate mixture of chi‐square distributions as the null distribution (Self & Liang, 1987) or assumed that the use of the chi‐square distribution with the appropriate number of degrees of freedom is conservative (Costa & Wilkinson‐Herbots, 2017; Self & Liang, 1987; Wilkinson‐Herbots, 2015). Because the currently available implementation of the above models allows the analysis of only two species at once, we prepared a separate data set for each pairwise comparison of *C. elongatoides*,* C. taenia*,* C. tanaitica* and *C. pontica*. The input data sets were represented by locus‐specific alignments of SNP positions with three rows–two rows with SNPs of both compared species and the third row containing SNPs of the *C. paludica* outgroup.

The coalescent models assume free recombination among loci but no recombination within loci and require three types of information for each pairwise species analysis. These consist of the number of nucleotide differences between pairs of sequences sampled (i) both from species 1, (ii) both from species 2 and (iii) one from each species. To estimate all parameters simultaneously, all three types of information must come from a different, independent set of loci (Wilkinson‐Herbots, 2012). Therefore, we randomly divided the analysed loci into three nonoverlapping data sets. The intraspecific data were simply calculated as the number of heterozygous positions at each locus, which in fact represents the number of nucleotide differences between a pair of alleles brought together by segregation into a sampled individual. The preparation of the third data set requires the comparison of two haploid sequences from different species, while our sequences originated from diploid individuals sometimes possessing multiple heterozygotic positions with unknown phase. We therefore extracted the longest possible alignment of SNPs where each compared individual (species 1 and species 2) had at most one heterozygous position by trimming the per‐locus alignments of SNPs. We then randomly selected one haploid sequence from each compared individual and recorded the number of nucleotide differences between them, along with the distance between the in‐ and outgroup, for the trimmed locus, to include information about relative mutation rates for each locus. To incorporate intrapopulation variability into our estimates, we sampled two individuals from each species and randomly sampled each locus from either one or the other individual (always keeping the sequence of SNPs within each locus from a single randomly selected individual).

The relative mutation rates at all loci were estimated by comparison with an outgroup species (Wang & Hey, 2010; Yang, 2002), *C. paludica*, whose divergence time from the ingroup was set to 17 Mya according to Majtánová et al. (2016). For each pairwise species comparison, estimated standard errors of the maximum‐likelihood estimates for the best‐fitting model were computed from the Hessian matrix. For species comparisons involving *C. elongatoides*, because the estimated standard errors of the migration rate are relatively large and because this parameter is of particular interest, we also computed 95% confidence intervals based on the profile likelihood, which are more accurate in the case of parameters near the boundary of the parameter space (Costa & Wilkinson‐Herbots, 2017; Pawitan, 2001), but which are computationally much more expensive to obtain.

### 2.4 Comparative analysis of genetic divergence between hybridizing fish species and types of reproductive isolation including hybrid asexuality

Finally, we tested the generality of the hypothesis that hybrids' asexuality represents an intermediate stage of the species diversification process by investigating the general association between the genetic divergence of hybridizing pairs of fish species and the dysfunction of their F1 fish hybrids. This analysis used data from Russell (2003), who investigated the association between the genetic divergence between hybridizing fish species (Kimura‐2‐parameter‐corrected distances in the cytochrome *b* gene) and the level of postzygotic isolation, which was categorized by an index ranging from 0 (both hybrid sexes fertile) to 4 (both sexes inviable). The index value 2 represented the stage when both hybrid sexes are viable but infertile, therefore preventing interspecific gene flow. We amended Russell's study by introducing an additional value (5) of postzygotic isolation index to those fish hybrids that have been documented to transmit their genomes clonally (gynogenesis, androgenesis) or hemiclonally (hybridogenesis). Altogether, the literature search performed led us to 17 cases of fish asexual hybrids (Appendix S4), which were added to the database of Russell (2003). In a single case, we modified Russell's (2003) data as he assigned *R. rutilus × A. bramma* hybrids a postzygotic isolation index of 0.5 but Slyn'ko (2000) showed that such hybrids produce clonal gametes and can reproduce via androgenesis. Therefore, *R. rutilus × A. bramma* hybrids were assigned the index value 5. In accordance with Russell's data, the cytochrome *b* gene divergence was calculated from available sequences of parental taxa using the Kimura‐2‐parameter (K2P) correction using the mega 5.0 asoftware (Tamura et al., 2011) (Appendix S4). The genetic distances of group 5 were compared with the other types of hybrids using the *t* test after the normality of the data was evaluated with the Shapiro–Wilk test.

Published cases of asexual hybrids concern nonoverlapping species pairs with three exceptions. *Cobitis elongatoides* has been involved in two crosses leading to naturally occurring asexuals (*C. elongatoides–taenia* and *C. elongatoides–tanaitica*), but as the original *C*. *tanaitica*‐like mitochondrion has been lost (see above), we considered the *C*. *elongatoides – C. taenia* cross only. *Hexagrammos octogramus* and *Poeciliopsis monacha* produce asexual hybrids by mating with two (*H. otaki* and *H. agrammus*) and three (*P. lucida*,* P. occidentalis*,* P. latipina*) congenerics, respectively. Hence, to avoid phylogenetic dependence, the analyses were repeated several times with only one cross *per* species.

## 3 RESULTS

### 3.1 Reproductive modes of Cobitis hybrids

#### 3.1.1 Reproductive modes of natural *C. elongatoides–tanaitica* hybrids

We successfully analysed backcrossed progeny of four natural EEN and one EN hybrid females. Progeny consistently expressed all maternal alleles, suggesting the production of unreduced gametes and lack of segregation. In one locus, a single progeny differed from the maternal allele by a single repeat, indicating a mutation event. A subset of the progeny also contained a haploid set of paternal alleles indicating that the sperm's genome is sometimes incorporated, leading to a ploidy increase (Tables 1 and S5). Comparison of allozyme profiles of eggs and somatic tissues of six EEN females revealed no evidence of premeiotic genome exclusion because allozyme profiles of all eggs were identical to the somatic tissues of the respective maternal individuals (Table S6), suggesting the absence of hybridogenesis.

#### 3.1.2 Reproductive modes of artificial F1 hybrids

*Cobitis elongatoides–taenia* hybrids were successfully backcrossed and analysed between 2004 and 2009 (Choleva et al., 2012). In addition, we successfully obtained two *C*. *taenia*–*C. pontica* primary crosses in 2006 (family Nos. 1 and 17 from which we ultimately obtained F2 progeny [Tables 1 and S5]). Both hybrid sexes were viable and fertile in both families No. 1 and No. 17, as evidenced by successful production of F2 progeny. F2 progeny mostly possessed one allele from the mother and the other from the father. Such inheritance patterns suggest the sexual reproduction of *C*. *taenia*–*pontica* hybrids in both families. However, family No. 1 also contained different types of F2 progeny: 15 individuals contained the complete set of maternal alleles. Nine of those individuals further possessed the haploid set of paternal alleles (Table 1). Such patterns indicate that *C*. *taenia–pontica* hybrid females produced both recombinant sexual and unreduced gametes.

### 3.2 Analysis of *C. elongatoides – C. tanaitica* hybrid zone and test of ongoing introgressive hybridization

**Figure 3:**
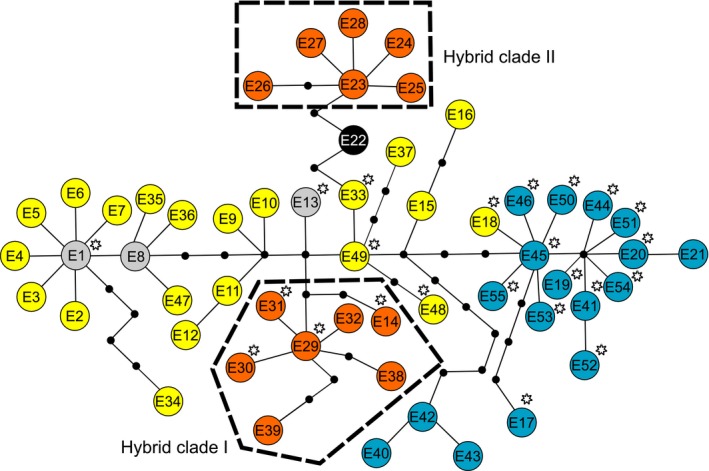
Median‐joining haplotype network showing phylogenetic relationships among *Cobitis elongatoides*‐like haplotypes of the cytochrome *b* gene. The network was constructed from previously published haplotypes and those from the current study (with asterisk). Yellow colour denotes haplotypes sampled in *C. elongatoides*; blue in *C. tanaitica*; black in *C. pontica*; orange in *C. elongatoides–tanaitica* hybrid (hybrid clade I) and *C. elongatoides–taenia* hybrid (hybrid clade II). Light grey circles denote haplotypes shared by both *C. elongatoides* and hybrids. Small black circles represent missing (unobserved) haplotypes

Eight hundred and eighteen *Cobitis* specimens were captured and identified from 26 localities all over the Lower Danubian River Basin. Genotyping by allozyme and PCR‐RFLP markers and flow cytometry revealed the presence of *C. elongatoides* at 11 sites and *C. tanaitica* at seven sites. Ten localities were inhabited by the distantly related species, *C. strumicae* (Figure 1 and Table S1). Apart from sexual species, we found various individuals that were heterozygotic in all species‐diagnostic loci indicating their hybrid state (Table S1). Most of them were polyploids, but we also encountered 31 diploid hybrids.

microchecker indicated two loci with potentially present null alleles, but none were consistent across populations; therefore, we did not exclude any locus from further analysis (Table S7). Summary statistics of microsatellite DNA analysis for three taxa are given in Table S8. Microsatellite analysis of 104 identified diploids (Table S7) altogether revealed that 28 of them clustered in eight groups of identical multilocus genotypes (MLG A—MLG H) with negligible probability of identical genotypes arising from independent sexual events (*p*_SEX_ **< 10^−5^). Comparison of pairwise distances against simulated distributions further revealed that several MLGs and/or individuals with unique genotype are related to each other with significantly lower genetic distances than would be expected from independent sexual events (*p *< .01). These were subsequently grouped into two MLL altogether indicating that 28 diploid individuals cluster in six distinct clonal lineages, each represented by two or more individuals. Notably, all such clonal individuals were identified as interspecific hybrids by the presence of diagnostic alleles in heterozygous states and by subsequent analyses. In addition, three hybrids had unique genotypes distant from any other individual, probably representing independent hybrid origins. No mtDNA haplotypes were shared by *C. elongatoides* and *C. tanaitica* (Figure 3). One clone (MLG B) possessed a haplotype E1, which was shared with *C. elongatoides*, while the remaining individuals assigned as hybrids possessed haplotypes clustering in the old hybrid clade I defined in Janko et al. (2005).

**Figure 4:**
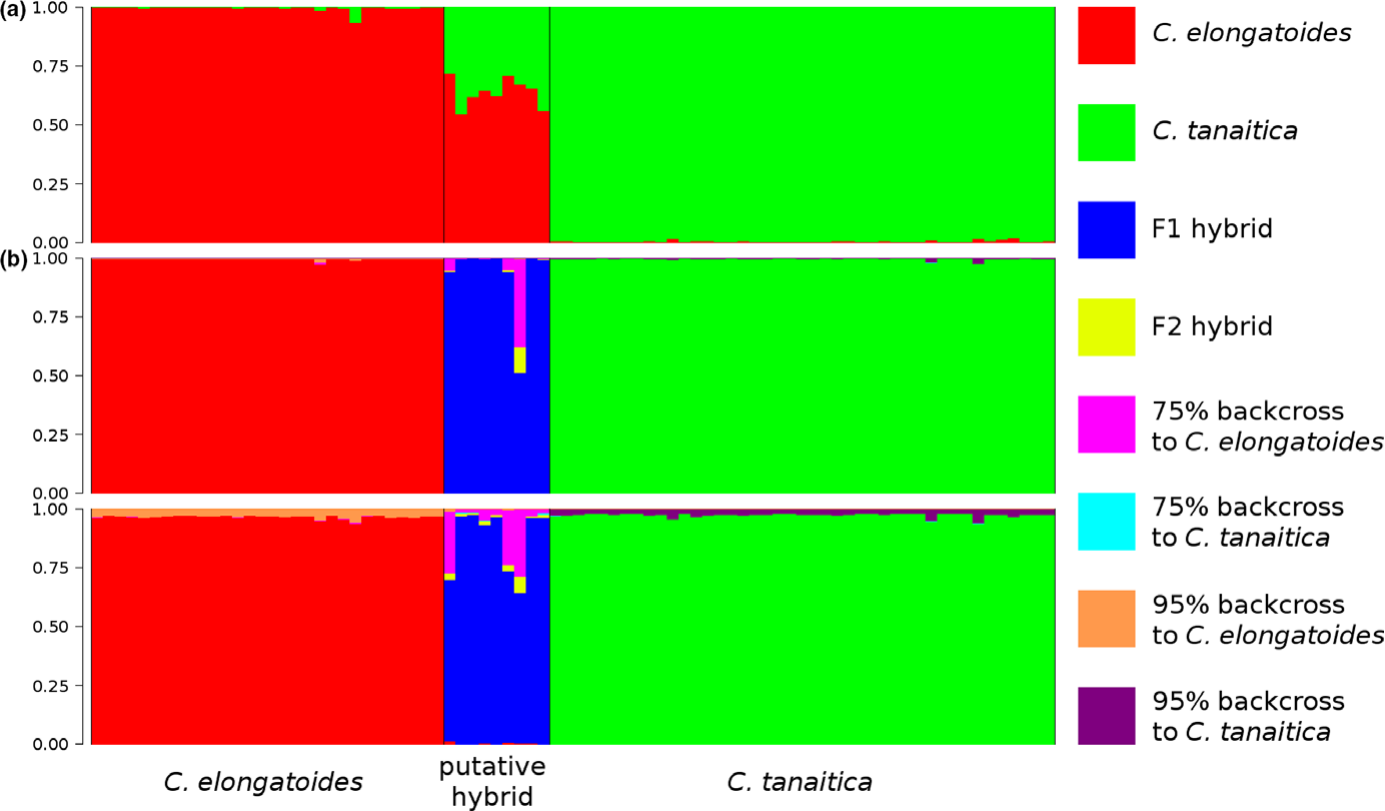
Population genetic analyses of the hybrid zone. (a) Individual proportion of membership to one of the two species‐specific clusters according to structure for *K* = 2. Each vertical bar represents one individual, and colours show the proportion of their assignment to the respective clusters corresponding to sexual species. For visual guidance, the individuals are grouped into a priori defined biotypes according to diagnostic allozyme markers (horizontal axis). (b) Classification of individuals' genotype according to NewHybrids. Each vertical bar represents one individual. Each colour represents the posterior probability of an individual belonging to one of the eight different genotypic classes. Individuals are sorted as in (a). The upper pane represents the results with Jeffreys prior and the lower pane with the uniform prior

Likelihood values provided by Structure converged during the runs, and results did not notably change between replicates. Although *Cobitis* specimens were sampled across many localities, Evanno et al.'s (2005) method implemented in StructureHarvester indicated *K *= 2 as the most likely number of genetic clusters for the diploid data set. Altogether, we found three types of diploid individuals, that is, those with the parameter *q* ranging between 0.003 and 0.019 and 0.934 and 0.997, respectively, presumably indicating pure *C. tanaitica* or pure *C. elongatoides* individuals, and those with intermediate *q* values (0.569–0.710; Figure 4a), which were identified as hybrids using our diagnostic markers (recall that only one representative of each clone was used for Structure input).

Data analysis using the NewHybrids software was slightly sensitive to the type of prior applied to Ɵ but not to π. However, consistent with Structure, all specimens presumed to be *C. elongatoides* and *C. tanaitica* were always assigned as the pure parental species (*p *> .95). Under Jeffrey's prior, NewHybrids assigned all but one of the above‐mentioned hybrids to the F1 class with probability exceeding 95%; however, in the case of MLL E, it could not distinguish between the F1 (*p = *.51), F2 (*p = *.11), and B1 (*p = *.37) states (Figure 4b). Under the uniform prior, most hybrids were assigned to the F1 class, but NewHybrids could not distinguish between the F1 and B1 states of MLL H, MLL E and individual 09BG19K22 (Figure 4b and Table S7).

### 3.3 Estimation of levels and timing of historical gene flow among species using exome‐capture data

After read mapping and SNP calling, we identified and removed from analysis potentially paralogous loci with spurious heterozygosity (see above) leaving us with the final data set of 52,473 SNPs from 6,192 contigs. Summaries of SNP variability for within‐ and among‐species comparisons are provided in Table S9.

Parent‐to‐offspring analysis confirmed the reliability of SNP detection showing that in >99% of SNPs, both experimental F1 progeny possessed variants derived from both parents and only less than 1% were potential mistakes (where F1s either possessed a new variant in a heterozygous state or, alternatively, a hybrid was called as a homozygote for one parental variant, while both parents were homozygotes for alternative alleles).

SNPs detected in wild‐caught animals were then analysed by fitting eight coalescent models assuming different scenarios of species divergence and connectivity (Table S3). The data sets of species pairs comparing *C. elongatoides* with either *C. taenia*,* C. tanaitica* or *C. pontica* were best fitted by models assuming isolation with initial migration (IIM7, followed by IIM8 as the second‐best model). The models of strict isolation (I7 and I4) provided a significantly worse fit than the IIM8 and IIM7 models, respectively (LRT *p* < .001 for all data sets). We also rejected the hypothesis of ongoing gene flow between *C. elongatoides* and other species as models assuming ongoing gene flow fitted the data poorly compared to models assuming isolation with initial migration. Specifically, IIM8 fitted all data sets significantly better than the nested IM5 model (LRT *p* ≪ 10^−10^) while the most complex GIM9 model did not significantly improve the fit compared to IIM7 or IIM8 (LRT *p* > .4 in all cases and evidence ratio <0.25). Furthermore, the parameter estimates obtained for the GIM9 model indicated a drastic decrease in recent migration rates compared to historical ones (*M*_c_ ~ 0), thus virtually converging to the IIM8 model. The GIM8 model assuming secondary contact could not be compared to the IIM7 or IIM8 models by LRT as these are not nested models, but its evidence ratio was low (<0.11 for all data sets) and it was significantly outperformed by GIM9 (LRT *p* < .05 in all cases), suggesting that *C. elongatoides* has been historically exchanging genes with the other species but became isolated in recent times.

The divergence time estimates were consistent across species comparisons. The results for the best‐fitting model (IIM7) indicated that *C. elongatoides* initially split from the other species roughly at 9 Mya but exchanged genes with them at estimated rates of between 0.05 and 0.1 migrant individuals per generation until time *t*_1_, for which ML estimates vary between 1.19 and 1.57 Mya depending on the data set (Table S3). Given that the speciation times of the other three species are much more recent (see below) than their split from *C. elongatoides*, our results suggest that the detected gene flow occurred predominantly between *C. elongatoides* and the common ancestor of the other three species.

For the pairwise data sets of the closely related *C. taenia*,* C. tanaitica* and *C. pontica*, the GIM9 model reduced to the IIM8 model as it gave 0 estimates for the current migration rate. The isolation‐with‐initial‐migration models (IIM7, IIM8) fitted the data significantly better than the isolation (I4 and I7) or isolation‐with‐migration (IM5) models (LRT *p* < .001 for all appropriate comparisons). However, the IIM models consistently suggested very intensive gene flow since *t*_0_ until a time *t*_1_ of approximately 0.15–0.3 Mya, at levels considered close to panmixia (e.g., Lowe & Allendorf, 2010), suggesting that those taxa probably formed a single perhaps substructured species until *t*_1_. Having modelled this scenario with the I6 model allowing a size change in the ancestral population, we obtained the best fit and estimated speciation time at around 0.3 Mya, further suggesting their recent speciation.

### 3.4 Comparative analysis of genetic divergence between hybridizing fish species and types of reproductive isolation including hybrid asexuality

**Figure 5:**
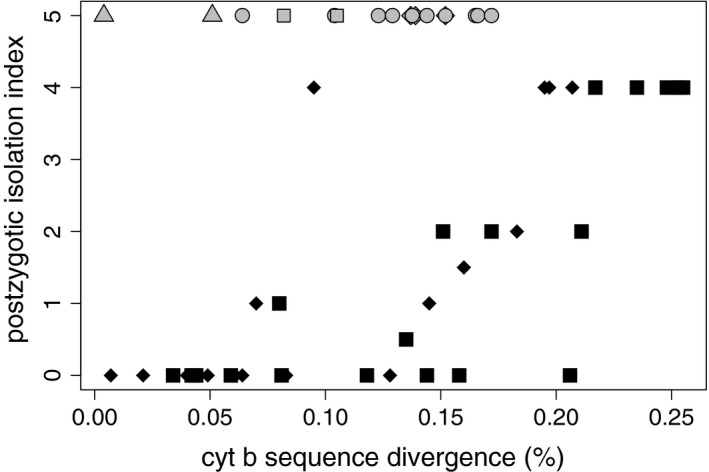
Plot of the postzygotic reproductive isolation index against K2P‐corrected distances in cytochrome b gene between hybridizing species. The reproductive isolation index is defined according to Russell's study as follows: 0, both hybrid sexes are fertile; 0.5, one sex fertile, the other sometimes infertile; 1, one sex fertile, the other infertile but viable; 1.5, one sex infertile but viable, the other sometimes still fertile; 2, both sexes viable but infertile; 2.5, one sex viable but infertile, the other sex only sometimes viable; 3, one sex viable, the other missing; 3.5, one sex sometimes viable, the other not; 4, both sexes inviable; 5, hybrids of at least one sex are known to form asexual lineages (highlighted in grey colour). Species pairs where one species occurred more than once in the analysis are indicated by grey triangles (*Cobitis taenia*), diamonds (*Poeciliopsis monacha*) and grey squares (*Hexagrammos octogramus*), respectively

Finally, we tested the generality of our findings, implying that the hybrids' asexuality may represent a transient stage of the species diversification process. To do so, we investigated the relationship between the genetic divergence of hybridizing pairs of fish species and the dysfunction of their F1 fish hybrids. We found that the K2P‐corrected divergences of cytochrome *b* gene sequences from fish species pairs that produce asexual hybrids range from 0.004 to 0.172 (mean *= *0.1192; *SE = *0.0459). This appears to be intermediate between those species pairs producing fertile and viable hybrids of both sexes (Russell's (2003) hybrid class 0; mean *= *0.079; *SE* *= *0.054) and those pairs that produce viable but infertile hybrids of both sexes (Russell's hybrid class 2; mean *= *0.179; *SE* *= *0.025) (Figure 5 and Appendix S4). Although some species of the *Cobitis*,* Hexagrammos* and *Poecilia* genera were involved in more types of hybridization producing asexuals, we included each species only once and repeated the analysis several times to account for all combinations. The Shapiro–Wilk test did not reject normality in any of the hybrid classes tested (*p *> .05). Regardless of the species pairs considered, parental divergences in the asexual hybrid class were always significantly lower than those of Russell's class 2 (Student's *t* test, *p *< .02) and always significantly higher than that of Russell's (2003) hybrid class 0 (*t* test, *p *< .03). Interestingly, the range of divergences among asexual hybrids is generally similar to the divergences of hybrids where the functionality of one sex is lower than that of the other (hybrid classes 0.5–1.5; mean *= *0.118; *SE* *= *0.040).

## 4 DISCUSSION

The principal role of hybridization in the evolution of asexuality is frequently debated, but the causality and underlying mechanisms are poorly understood (Dijk, 2009; Kearney et al., 2009). Our study not only provides clear support for earlier hypotheses that emergence of asexuality correlates with the divergence between parental species, but it also offers a conceptually novel view suggesting that hybrid asexuality forms an inherent stage of the process of species diversification with possible effects on the speciation itself.

### 4.1 Accomplishment of speciation in spite of fertile hybrids

Two lines of evidence indicated the absence of interspecific gene flow between *C. elongatoides* and both its parapatric counterparts *C. taenia* and *C. tanaitica*, suggesting the accomplishment of speciation. Experimental crossings (Choleva et al., 2012; Janko, Bohlen, et al., 2007; this study) showed that *elongatoides – taenia* and *elongatoides–taenia* hybrid females are fertile but do not produce reduced gametes neither through the “standard” sexual process, nor by genome exclusion (hybridogenesis). Instead, hybrid females produce unreduced gametes that either develop clonally or occasionally incorporate the sperm's genome leading to polyploidy, but are unlikely to enable interspecific gene flow. Hybrid males also do not appear to mediate gene flow as *C. elongatoides–taenia* hybrid males are infertile (Choleva et al., 2012) and *C. elongatoides–tanaitica* hybrid males have not been observed in nature.

Similar conclusions are drawn from analyses of hybrid zones between *C. elongatoides* and *C. taenia* or *C. tanaitica*. Both zones revealed parallel evolutionary patterns being dominated by polyploid asexuals (in the Danube drainage, they also invaded rivers inhabited by phylogenetically distant species *C. strumicae* (Choleva et al., 2008)). Diploids were less frequent, and unlike classical hybrid zones, where the genetic admixture follows a continuum, we found a trimodal distribution of the *q* parameter indicating the presence of both pure species (*q* ~ 0 or 1, respectively) and their hybrids with intermediate values. Most diploid hybrids apparently formed clonal lineages and mtDNA analysis indicated a prevalently unidirectional origin of asexuality within both zones; *C. elongatoides* was maternal to all Danubian hybrids, while *C. taenia* was maternal to the vast majority of *elongatoides–taenia* clones (Janko et al., 2012).

The detected hybrids were assigned as F1 (NewHybrids software, *p *> .95) except three Danubian diploid lineages where NewHybrids could not reject the B1 state although the F1 state was still preferred. Interestingly, these three lineages belong to the old clonal hybrid clade I and possess a number of private microsatellite alleles, suggesting that their assignment by both the Structure and NewHybrids software might have been affected by mutational divergence from contemporary sexual species (see, e.g., da Barbiano, Gompert, Aspbury, Gabor, & Nice, 2013). We are aware that NewHybrids may fall short in detecting backcrosses using a limited number of loci especially when between‐species divergence is low (Vähä & Primmer, 2006). However, our inferences are unlikely to have been substantially affected by this problem because interspecific *F*_ST_ values were large in all loci and the observed lack of resolution did not concern misclassification between B1/pure species as suggested by Vähä and Primmer (2006) but rather between the B1 and F1 classes.

The absence of recombinant hybrid progeny altogether agrees with crossing experiments and implies the lack of introgressive hybridization between *C. elongatoides* and other species. Interestingly, the isolation of their gene pools appears to be driven neither by strong prezygotic RIMs nor by extrinsic postzygotic isolation, as species readily hybridize both in natural and experimental conditions (Choleva et al., 2012; Janko, Flajšhans, et al., 2007; Janko et al., 2012), and do not notably differ in ecological requirements. Instead, the data show that ultimately, a reproductive barrier separating the gene pools of contemporary species is ensured by asexual reproduction of hybrid females and sterility of hybrid males.

### 4.2 Historical periods of gene flow and change in hybrids' reproductive mode

Despite current postzygotic isolation, hybrids able to mediate gene flow must have existed in the past as indicated by the coalescent analysis, where the isolation‐with‐initial‐migration models outperformed both the strict isolation and isolation‐with‐migration models, for all pairwise species comparisons involving *C. elongatoides*. The best‐fitting IIM models suggested historical episodes of gene exchange after the initial divergence of *C. elongatoides* around 9 Mya, followed by isolation of contemporary species since *t*_1_, estimated at around 1–1.5 Mya (Figure 2 and Table S3). While the models only allowed pairwise species comparisons, the results indicated significant historical gene flow between *C. elongatoides* and each of the other three species. As these three species diversified only recently (~0.3 Mya), it is reasonable to conclude that the inferred gene flow occurred predominantly between their common ancestor and *C. elongatoides* although we may not rule out that the gene flow continued also after the *C. taenia – C. tanaitica–C. pontica* speciation.

The present results are consistent with a previous application of a Bayesian IM model to nine nuclear and one mtDNA loci (Choleva et al., 2014) but two differences were noted. First, Choleva et al. reported significant mitochondrial but not nuclear *C. elongatoides – C. tanaitica* gene flow, which led to the impression that the nucleus has not been affected by hybridization despite complete introgressive replacement of *C. tanaitica*'s mitochondrion. The current evidence for gene flow also in the nuclear compartment is more plausible biologically. Second, the present data indicate isolation among the three closely related species while the previous study suggested intensive *C. taenia–C. tanaitica* gene flow. This was at odds with the field data because sympatry between both species has not been documented and we never observed their hybrids in nature (Janko, Flajšhans, et al., 2007). The present analysis is therefore again more in line with current knowledge about *Cobitis*. The discrepancy with the previous inference may potentially reflect the tendency of Bayesian IM algorithms to inflate estimates of gene flow when the number of loci is low and splitting times are recent (Cruickshank & Hahn, 2014; Hey, Chung, & Sethuraman, 2015). Given that crossing experiments indicated fertility of hybrids between closely related species, their genetic isolation may potentially result from the distribution in separated inflows of the Black Sea, rather than from other pre‐ or postzygotic RIMs. In any case, more intensive sampling and analyses are required to fully understand the diversification of these close relatives.

As with all models, various violations of assumptions might have affected our inferences. These include uncertainty regarding the relative mutation rates of the different loci, population size fluctuations, intralocus recombination and geographical structure. Model results might further have been affected by direct or background selection (Walczak, Nicolaisen, Plotkin, & Desai, 2012). Moreover, the intensity of gene flow was probably not constant between *t*_0_ and *t*_1_, and pairwise coalescent analyses might have been affected by intractable interactions with other species although IM models are reasonably robust to this type of violation (Strasburg & Rieseberg, 2010).

However, although our model‐based inference might have suffered from various such limitations, we emphasize that independent types of data corroborated the scenario of historical introgressive hybridization with little or no contemporary gene exchange between *C. elongatoides* and the other species. On the one hand, the existence of intensive gene flow between *C. elongatoides* and the other species is supported by the massive introgression of its mitochondrial lineage into *C. tanaitica (*Choleva et al., 2014). On the other hand, the lack of contemporary gene flow is evidenced by small but nonzero divergences between contemporary *C. elongatoides* mtDNA haplotypes and those fixed in *C. tanaitica* as well as by clonal reproduction of hybrids and the apparent lack of introgressive hybridization in hybrid zones. Finally, the crossing experiments indeed showed that recently diverged species pairs are able to produce mostly fertile and recombining F1 and F2 progeny of both sexes (*C. taenia* and *C. pontica* crosses from this study), while hybrids between substantially diverged species cannot mediate gene flow, being exclusively represented by sterile males and highly fertile, yet clonal females (*C. elongatoides* and *C. taenia* crosses by Choleva et al., 2012).

### 4.3 Simultaneous evolution of asexuality and RIMs

Several complementary approaches consistently indicated that the incipient spined loach species originally produced hybrids whose reproductive mode enabled more or less intensive gene flow, but as those species diverged further, introgressions became restricted and asexuals were the major type of hybrids. Hybrid asexuality arose multiple times in the family *Cobitidae*, involving *taenia* – *pontica* crosses (laboratory hybrids from this study), *elongatoides* – *taenia* and *elongatoides* – *taenia* crosses (Choleva et al., 2012; Janko, Flajšhans, et al., 2007) as well as two cases from other genera in Asia (Kim & Lee, 2000; Zhang, Arai, & Yamashita, 1998). Such a widespread and independent emergence of asexuality is not consistent with the phylogenetic‐constraint hypothesis (Hotz et al., 1985; Murphy et al., 2000). It rather conforms to the balance hypothesis (Moritz et al., 1989), which predicts that hybridization between gradually diverging species would initially produce mostly sexual hybrids while successful asexuals would arise at intermediate stages when a hybrid's meiosis is disrupted but fertility is not yet significantly reduced. Simultaneously, the inferred diversification history of spined loaches is consistent with the gradual decline in the species' capability of introgressive hybridization that is expected to evolve along the speciation continuum from weakly separated entities towards strongly isolated species (e.g., Seehausen et al., 2014).

The establishment of hybrid asexuality thus appears in many aspects similar to the gradual accumulation of intrinsic postzygotic RIMs. Most notably, both processes correlate with the divergence of hybridizing species. It is interesting to note that the interval of divergences allowing the initiation of clonality may be wide as successful clones readily emerge from hybridization between *C. elongatoides* and other species, diverged ~9 Mya, while we also documented asexuality between recently diverged *C. taenia* and *C. pontica*. Furthermore, analogously to RIM accumulation, the establishment of hybrid asexuality appears asymmetrical with respect to the direction of cross (Janko, Kotlik, & Ráb, 2003; Wirtz, 1999) as well as the sex of hybrids—asexuality is present in females, while hybrid males in most species combinations are sterile. Although extensive investigation of *Cobitis* sex chromosomes is yet to be carried out, two studies indicated male heterogamety in Asian *C. striata* and European *C. tanaitica* (Saitoh, 1989; Vasil'eva & Vasil'ev, 1998), suggesting that our data conform to the empirical observation that the heterogametic sex tends to acquire infertility earlier than the homogametic one (Bolnick & Near, 2005; Haldane, 1922; Russell, 2003). Such analogies with postzygotic RIMs justify the hypothesis that hybrid asexuality may be considered a specific form of Dobzhansky–Muller incompatibility evolving during the species diversification process.

The results obtained by adding asexual hybrids into Russell's (2003) comparative analysis suggests that the scenario revealed in spined loaches has the potential to be generalized. Asexual hybrids appear at higher levels of parental divergences than species pairs producing fertile and viable hybrids but lower than those producing infertile hybrids of both sexes (Figure 5, Appendix S4). Similar patterns were observed also in reptiles (Jančúchová‐Lásková et al., 2015; Moritz, Densmore, et al., 1992; Moritz, Uzzell, et al., 1992; Moritz, Wright, et al., 1992). The divergence of asexual‐producing species pairs is similar to those producing hybrids with lowered hybrid fertility in one sex (Russell's hybrid classes 0.5–1.5), which is in line with the observation that populations of asexual fish often consist of females only. The relatively wide interval of divergences allowing the initiation of asexuality (Figure 5) also agrees with the expectation that accumulation of RIMs follows a variable‐rate clock and that different types of incompatibilities accumulate in a noisy manner (Edmands, 2002).

We may now ask whether hybrid asexuality may represent the true primary barrier, thereby directly contributing to speciation, or only a way to temporarily rescue hybrids that would otherwise be sterile anyway. As the species diverge, meiosis and gametogenesis become impaired in hybrids (Russell, 2003; Sánchez‐Guillén et al., 2014) leading to sterility and gene flow reduction. Some organisms, like spined loaches, might have temporarily alleviated sterility by making fertile, yet asexual, hybrids. Possibly, if *Cobitis* hybrids were unable of asexuality, they would be sterile and gene flow would occur at the same low rate as in the actual situation. Hybrid asexuality would thus have no specific effect on speciation. However, *Cobitis* asexual females, just like many other hybrid asexuals, are highly fertile and retained functional meiosis (the clonality is achieved by premeiotic endoduplication followed by two meiotic divisions (Saat, 1991; Juchno, Arai, Boroń, & Kujawa, 2016). Moreover, hybrid asexuality generally appears at lower divergences than hybrid sterility (Figure 5). We therefore prefer the alternative hypothesis viewing hybrid asexuality as a true form of postzygotic RIM that evolves earlier in the diversification process than sterility.

## 5 CONCLUSIONS

Our data suggest that hybrid asexuality constitutes a transient stage of the speciation continuum that may be viewed as a special case of Dobzhansky–Muller incompatibilities and tends to evolve at lower divergence than hybrid sterility or inviability. Given that asexuals are unlikely to mediate gene exchange, the production of asexual rather than sexual hybrids may help to establish an effective barrier even in the absence of other typical forms of postzygotic RIMs. This implies a possibility that various currently incompatible species might have historically produced asexual hybrids that have gone extinct. Such a phase might be difficult to detect from current patterns due to its transiency and the typically short lifespan and cryptic nature of asexual lineages (Butlin, Schön, & Martens, 1999).

In any case, it appears that there is a mechanistic connection between recombination suppression, hybrid incompatibility and speciation (Balcova et al., 2016). Given that asexuality is one of the most prominent processes that alter recombination, the stage of hybrid asexuality requires close attention as it might represent an underappreciated mechanism in the speciation of those groups which are capable of asexual reproduction, including arthropods, vertebrates or plants.

## DATA ACCESSIBILITY

The supplemental Tables, Appendices S1–S9 and raw data of rna‐seq and exome‐capture sequence data are available from the Dryad Digital Repository: https://doi.org/10.5061/dryad.7nt8f.

## AUTHOR CONTRIBUTION

K.J. formulated the central hypothesis of the study and drafted the manuscript; K.J., J.P. and L.C. conceived and designed the experiments; K.J., N.I., J.K., J.Ri., R.R., V.Š., M.H. and L.C. performed the experiments; K.J., J.P., H.W.H., R.J.C., P.D., J.K., J.Ro. and L.C. analysed the data; H.W.H. and R.J.C. developed the mathematical models; K.J., H.W.H., J.K., R.R. and L.C. contributed to the MS writing.
